# Study protocol – A systematic review and meta‐analysis of hypothermia in experimental traumatic brain injury: Why have promising animal studies not been replicated in pragmatic clinical trials?

**DOI:** 10.1002/ebm2.20

**Published:** 2016-10-18

**Authors:** Theodore C. Hirst, Ralf Watzlawick, Jonathan K. Rhodes, Malcolm R. Macleod, Peter J.D. Andrews

**Affiliations:** ^1^ Centre for Clinical Brain SciencesUniversity of EdinburghEdinburghUK; ^2^ Department of NeurosurgeryRoyal Victoria HospitalBelfastUK; ^3^ Department of Neurology and Experimental NeurologyCharité Campus Mitte, Charité‐UniversitätsmedizinBerlinGermany; ^4^ Department of Critical Care, Anaesthesia & Pain MedicineUniversity of EdinburghEdinburghUK

**Keywords:** hypothermia, meta‐analysis, systematic review, traumatic brain injury

## Abstract

Traumatic brain injury (TBI) is a major cause of death and permanent disability. Systemic hypothermia, a treatment used in TBI for many decades, has recently been found to be associated with neutral or unfavourable clinical outcomes despite apparently promising preclinical research. Systematic review and meta‐analysis is a tool to summarize literature and observe trends in experimental design and quality that underpin its general conclusions. Here we aim to use these techniques to describe the use of hypothermia in animal TBI models, collating data relating to outcome and both study design and quality. From here we intend to observe correlations between features and attempt to explain any discrepancies found between animal and clinical data. This protocol describes the relevant methodology in detail.

## 1 BACKGROUND

Traumatic brain injury (TBI) represents a significant challenge in healthcare across the world: it is the leading cause of death and permanent disability in young adults and incidence is increasing.^2^, ^3^ Despite significant progress in the understanding of pathophysiology and developments of novel experimental treatments, there have been no consequential therapies successfully translated into clinical practice. Similarly, many of the treatments accepted as standard care have weak evidence bases.^4^, ^5^, ^6^, ^7^

Hypothermia is a therapy well established in the neuro‐intensive care, having been commonplace in TBI management for half a century. It is based on the underlying principle that hypothermia controls dangerously elevated intracranial pressure and mediates damage to neural tissue from hypoxic and other metabolic mechanisms. However there is increasing evidence that, in TBI patients, induced hypothermia is at least as effective at controlling intracranial pressure as other medical therapies, however, importantly it is associated with neutral or unfavourable long term outcomes.^4^, ^8^, ^9^

These findings contradict a consensus that hypothermia in *in vivo* studies is effective. We therefore seek to describe the preclinical literature using systematic review and meta‐analysis, and aim to provide an explanation as to why this discrepancy might exist. We hypothesize that the preclinical dataset will consist of a large number of small, heterogeneous studies with differences in efficacy associated with features relating to both risk of bias (randomization, blinding, publication bias) as well as experimental design features (features of animals used, TBI model, hypothermia treatment and control group temperature).

## 2 METHODS

### 2.1 Literature search

We will search PubMed, MEDLINE and EMBASE using the search strategies below:

(traumatic brain injury OR TBI OR head injury OR head trauma OR brain trauma OR neurotrauma OR cortical trauma OR cerebral trauma OR weight drop OR fluid percussion OR controlled cortical impact OR projectile concussive impact OR blast‐induced neurotrauma) AND (hypotherm* OR normotherm* OR temperature OR thermoregulat* OR cool* OR cold OR chill OR cryo*)

Searches will be limited to animals using previously developed filters.^10^, ^11^

### 2.2 Study selection

We will include studies that satisfy the following inclusion criteria:

1. Animal model, non‐neonatal
2. Non‐penetrating traumatic brain injury model
3. Systemic hypothermia used as monotherapy
4. Compared to normothermic or hyperthermic control in same study
5. Mean / Median value, variation (SE, SD, CI) and the number of animals stated for each group
6. All outcomes relating to neurobehavioural assessment, ICP, contusion size, mortality, biochemical or histological markers of neuronal cell death, axonal damage, oedema

We will first screen titles and abstracts, excluding studies not relating to an animal model of TBI. Subsequently we will screen full articles of remaining studies and apply the above selection criteria. Two authors will screen studies (TCH/RW) and differences will be resolved by discussion. In cases of no consensus and third reviewer (MRM) will be consulted.

There will be no language or date restrictions and all peer review articles, conference abstracts and other modalities of publication will be accepted if sufficient information is provided for inclusion. Reviews and clinical papers will be excluded.

### 2.3 Methodological quality and study bias

We will assess risk of bias according to a modified scale developed by the CAMARADES group for stroke studies:^12^

1. Peer reviewed journal
2. Randomized group allocation with method stated
3. Blinded induction of injury
4. Blinded assessment of outcome
5. Sample size calculation
6. Statement of potential conflict of interest
7. Animal welfare policy stated
8. Use of anaesthetic agent without intrinsic neuroprotective property (ketamine)
9. Reporting of total number of animals treated
10. Explanation of excluded animals

### 2.4 Data extraction

We will use neurobehavioural outcome scores as our primary outcome.

Secondary outcome measures: we will include pathological indicators such as contusion/haematoma size, physiological measurements such as intracranial pressure and biochemical markers of oedema, cell death or axonal damage.

We will include all studies comparing a hypothermic treatment group (induced or permissive) with a normothermic or hyperthermic control (induced or natural disease course). For studies reporting more than one group we will include all comparisons, correcting for the number of control groups as appropriate.

Relating to study design, we will extract the information outlined in Table 2.

**Table 1:** Progress at time of protocol submission

Stage of process	Started	Completed
Preliminary searches	Yes	Yes
Piloting study selection	Yes	No
Formal screening with final search criteria	No	No
Data extraction from included papers	No	No
Quality assessment	No	No
Data analysis	No	No
Manuscript writing	No	No

**Table 2:** Study design characteristics to be extracted

Study identifiers	Author Year of publication Journal
Animal population	Species Strain Comorbidities Sex Age
TBI paradigm	Injury model type (weight drop, fluid percussion, controlled cortical injury, projectile concussive impact, penetrating ballistic‐like brain injury, blast‐induced neurotrauma) Cranium unopened/craniotomy/craniectomy Location of injury (lobe) Impactor velocity/peak pressure wave Baseline neurobehavioural score Method of animal head stabilization Anaesthetic agent Anaesthesia duration
Hypothermia	Target temperature Duration of hypothermia Method of hypothermia induction (intra/extracorporeal, permissive) Delay to treatment Rate of rewarming Method of rewarming Control group temperature 1) normothermia (36.0‐37.9°C for mammals) or 2) hyperthermia (≥38.0°C) Control group temperature induced or passive
Primary outcome measure	Neurobehavioural outcome score
Secondary outcome measures	Change in contusion size ICP Mortality Biochemical or histological markers: oedema (eg AQP4), axonal damage (e.g. APP), neural cell death

### 2.5 Data collation and analysis

Data will be extracted in duplicate by TCH and RW. Differences in extracted data will be resolved by discussion. We will extract data from publications via text, if available, or by measurement from graphs with a digital screen ruler. Should this information not be immediately clear from the publication we will contact the authors directly. We will correct for the multiple use of a single control group by dividing the number of animals in each control group by the number of times represented in the dataset.

For neurobehavioural outcomes, we will pool data for analysis using the DerSimonian and Laird random‐effects model, as we expect a large degree of methodological variability between studies, and assess for the presence of heterogeneity using the I^2^ statistic. We will then attempt a multi‐variable metaregression to investigate relationships between reported efficacy and the study quality and design features highlighted above, for variables in which there are 10 studies or more in every group. In sensitivity analysis, we will perform a univariate metaregression.

For secondary outcomes, namely contusion size, ICP, mortality and biochemical markers we intend to first perform a frequency analysis to describe the number of times each outcome or molecule is reported in the literature. Following this, we will calculate efficacy estimates (via a random‐effects model) for those outcomes with 10 or more included experiments. We have set this threshold as this is a level at which we deem there to be enough data to warrant collation for meta‐analysis.

We will search for publication bias using funnel plots, Egger regression and p‐curve analysis.^13^

Changes in this analysis plan will be announced and reported in the study publication subsequently.

### Conflict of interest

The authors declare that there are no conflicts of interest.
